# Poor Subjective Sleep Quality Predicts Symptoms in Irritable Bowel Syndrome Using the Experience Sampling Method

**DOI:** 10.14309/ajg.0000000000002510

**Published:** 2023-09-22

**Authors:** Rabia Topan, Lisa Vork, Heather Fitzke, Shraya Pandya, Daniel Keszthelyi, Jan Cornelis, Jason Ellis, Lukas Van Oudenhove, Maaike Van Den Houte, Qasim Aziz

**Affiliations:** 1 Blizard Institute, Wingate Institute of Neurogastroenterology, Centre for Neuroscience, Surgery and Trauma Barts and the London School of Medicine and Dentistry, Queen Mary University, London, UK;; 2 Division of Gastroenterology-Hepatology, Department of Internal Medicine, Maastricht University Medical Center, NUTRIM School of Nutrition and Translational Research in Metabolism, Maastricht University, Maastricht, the Netherlands;; 3 Centre for Medical Imaging, University College London;; 4 Imec, Kapeldreef 75, Heverlee, Belgium;; 5 Northumbria Centre for Sleep Research, Department of Psychology, Northumbria University, UK;; 6 Laboratory for Brain-Gut Axis Studies (LaBGAS), Translational Research in Gastrointestinal Disorders (TARGID), Department of Chronic Diseases & Metabolism (CHROMETA), KU Leuven, Leuven, Belgium;; 7 Leuven Brain Institute, KU Leuven, Leuven, Belgium;; 8 Cognitive & Affective Neuroscience Lab, Department of Psychological & Brain Sciences, Dartmouth College Hanover, New Hampshire, USA.

**Keywords:** sleep, irritable bowel syndrome, experience sampling method, actigraphy

## Abstract

**INTRODUCTION:**

Sleep quality may affect symptom experience in irritable bowel syndrome (IBS). Our aim was to investigate the relationship between sleep quality and gastrointestinal (GI) symptoms using actigraphy and the experience sampling method.

**METHODS:**

Patients with IBS were recruited from a tertiary Neurogastroenterology clinic and the community. GI symptoms and mood were recorded on a smartphone application, 10 times per day, over 7 consecutive days. Subjective sleep quality was recorded every morning to reflect the night before. Objective measures of sleep quality were estimated from wrist-worn actigraphy. Cross-lagged structural equation models were built to assess the directionality of sleep-symptom relationships over time.

**RESULTS:**

Eighty patients with IBS completed the study (mean age: 37 years [range 20–68], 89% female, 78% community). Approximately 66% had a Pittsburgh Sleep Quality Index score ≥ 8, indicating a clinically significant sleep disturbance. Approximately 82% (95% CI: 72–90) screened positive for a sleep disorder, most commonly insomnia. In cross-lagged analysis, poor subjective sleep quality predicted next-day abdominal pain (0.036 < *P* < 0.040) and lower GI symptoms (0.030 < *P* < 0.032), but not vice versa. No significant relationship with GI symptoms was found for any objective sleep measure using actigraphy.

**DISCUSSION:**

Poor subjective sleep quality was associated with higher next-day lower GI symptom levels, but not vice versa. Objective sleep measures did not predict next-day abdominal symptoms, potentially supporting the conclusion that it is the perception of sleep quality that is most influential. This study may be used to guide future research into the effect of sleep interventions on GI symptoms.

## Graphical abstract

**Figure:**
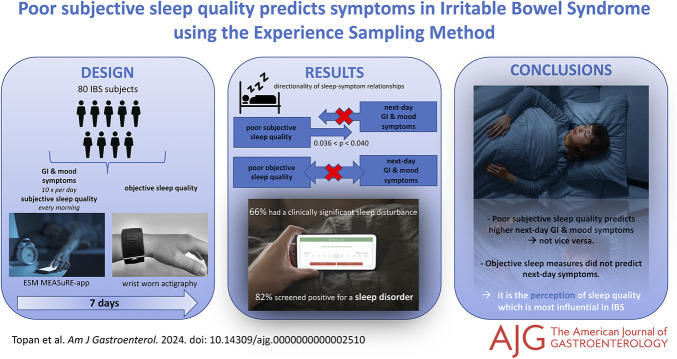

## INTRODUCTION

Sleep disorders are more common in patients with irritable bowel syndrome (IBS) compared with those in healthy subjects with a pooled prevalence of 37.6% (1,2). On average, patients with IBS sleep more hours per day but feel less well rested compared with healthy controls (3). However, only 2.4% of patients attribute their sleep disturbances to gastrointestinal (GI) symptoms.

Associations between GI symptoms and sleep quality in patients with IBS have been investigated before. Patel et al (3) found that waking episodes during sleep, measured objectively, were associated with greater abdominal pain and lower general and IBS-specific quality of life. Buchanan et al (4) concluded that poor subjective sleep quality predicts next-day abdominal pain, but not other GI symptoms, and objectively measured sleep efficiency significantly predicts next-day anxiety and fatigue but not abdominal pain. Furthermore, GI symptoms measured by end-of-day diaries did not predict subsequent sleep quality suggesting that sleep is an independent factor affecting GI symptoms (4). Yet most studies are limited by small sample sizes and fail to assess the directionality of effects between specific sleep quality measures and specific symptoms.

Given the well-established association between mood disturbance and IBS (5), the effect of comorbid anxiety and depression are worth considering when investigating the role of sleep-symptom relationships. Previous studies suggest that anxiety symptoms during the day may predict time taken to fall asleep because hypervigilance and rumination are psychological processes that are likely to delay sleep onset (6). Whereas depression has been linked to an increased likelihood to awaken early in the morning (7).

IBS symptom monitoring has traditionally been conducted via single time point, retrospective questionnaires, which by nature are prone to recall bias (8), fail to account for within-day symptom variability (9), and cannot be used to decipher the directionality between symptoms and the factors that influence them. The experience sampling method (ESM) has been used in IBS to overcome these limitations by quantifying symptom burden repeatedly and randomly throughout the day (10). ESM has demonstrated real-time associations between abdominal pain and stress, underlining the importance of day-to-day variability and longitudinal relationships when interpreting drivers of GI symptoms in IBS (11).

Subjective sleep measures include validated questionnaires and sleep diaries; while objective measures include polysomnography (PSG)—a component of which is comparable with actigraphy (12). An actigraph is an accelerometer worn by a subject to record movements (acceleration [ACC]), the nocturnal portion of which is then used to estimate sleep-wake states (13). A study by Rotem et al (14) reports that patients with IBS experience impaired sleep quality and significant sleep fragmentation on PSG, which was supported by actigraphy findings. By contrast, Elsenbruch et al (15) found that patients with IBS had significantly increased scores on the Pittsburgh Sleep Quality Index (PSQI) compared with healthy subjects, but no significant group differences on PSG. It is widely accepted that there is a mismatch between subjective reporting of sleep quality and objective measurements, which is described as sleep state misperception (16). Studies have yet to conclude whether it is *objective* or *subjective* sleep quality that is associated with GI symptoms in the population with IBS, and this is hampered by lack of consistency between studies on the sleep measures they use.

In the current literature, studies addressing the relationship between sleep quality and GI symptoms remain limited by small sample sizes, recall bias, heterogeneity between sleep quality measures, and perhaps most importantly, the failure to address directionality of relationships in daily life in a single comprehensive model.

The aim of this study was therefore to evaluate the directionality of relationships between subjective sleep quality, objective sleep measures, and GI symptoms in a well-defined population with IBS using actigraphy and ESM as real-time, repeated measurement methods.

## METHODS

### Study design

**Figure 1.**
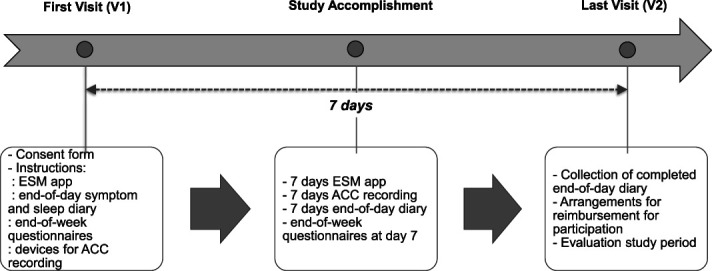
Study design. ACC, accelerometer; ESM, electronic sampling method; V1, visit 1; V2, visit 2.

Patients with IBS were recruited between March 2020 and June 2021 from the tertiary Neurogastroenterology clinic at The Royal London Hospital, and from the community, in London, UK. The study protocol has been approved by the South Central—Hampshire A Research Ethics Committee (REC reference 19/SC/0236). Data was collected during 7 consecutive days, and a time line of the study period is shown in Figure 1.

### Study patients

We accessed the community group through social media campaigns and the IBS network, a registered charity for patients with IBS. Inclusion criteria comprised the following: aged 18–70 years; IBS diagnosed according to Rome IV criteria (17), including subtype assessment, by a trained clinical researcher on the first study visit; ability to give informed consent; understand and speak English; and access to smartphone technology. Exclusion criteria comprised the following: any organic explanation for symptoms; history of abdominal surgery (aside from uncomplicated appendicectomy, cholecystectomy, and/or hysterectomy); new or dose change of regular medication/supplement commenced within the last 1 month; and/or known allergy to silicone-based adhesives/prior skin condition, e.g., severe atopic eczema.

### Symptom measures

The following measures were used to report on the directionality of sleep-symptom relationships: subjective sleep and GI symptom measures, objective symptom measures alongside sleep diary-because they were all measured in real time. In addition, we measured GI and sleep symptoms at the end of the 7-day study period, which were used for descriptive purposes only.

### Subjective sleep and GI symptom measures

**Figure 2.**
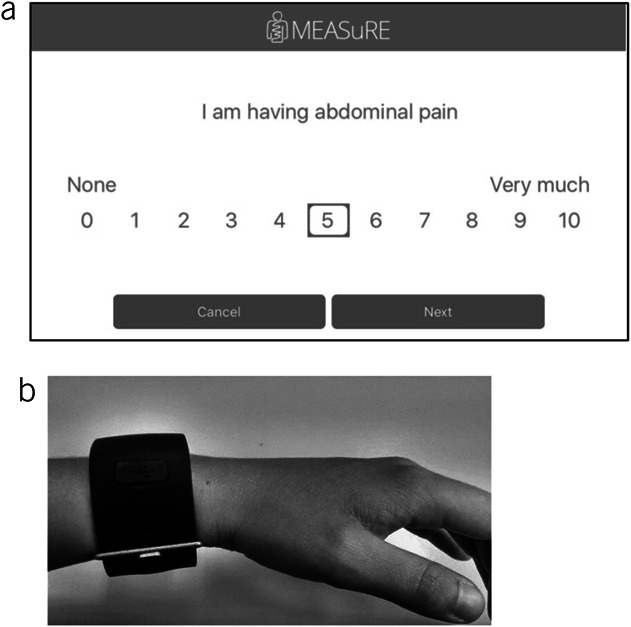
(**a**) Screenshot of the ESM question for the assessment of abdominal pain; (**b**) wristband (3D accelerometer).

To use ESM, patients were instructed on how to use a digital application (Maastricht Electronic Abdominal Symptom REcording [MEASuRE]) on their smartphone, specifically developed for this purpose (10). The application sends text notifications alongside an auditory signal, 10 times per day, at random moments, between the hours of 07:30 and 22:30 with a minimum of 15 minutes and maximum of 3 hours between subsequent signals. The notification prompted completion of an ESM questionnaire, which included the same questions each time, over 7 consecutive days, designed to assess the following: abdominal pain, upper- and lower-GI symptoms, anxiety, depression, and nocturnal abdominal problems. Each question was scored on an 11-point numeric rating scale (0 = not at all to 10 = very severely) (18,19). After each notification, the ESM questionnaire remained available for a 10-minute window, after which it was logged as missing data when not completed. A screenshot of the MEASuRE application for the item abdominal pain is shown in Figure 2a.

Subjective sleep quality was reported using the MEASuRE app in the morning—to reflect the night before—including the extent to which subjects slept well (*SleptWell*) scored from 0 (none) to 10 (very much); time taken to fall asleep (*SleepOnsetLatency*_subjective_) and time lying in bed awake before getting out of bed (*EarlyMorningAwakening*_*subjective*_)—scored from 0 to 5 minutes, 5–15 minutes, 15–30 minutes, 30–45 minutes, 45 minutes–1 hour, 1–2 hours, and more than 4 hours; and finally, number of awakenings in the night (*NumberofAwakenings*_*subjective*_) scored from 0 to >5 times.

### Objective sleep measures

Patients were sent a wristband alongside a charging cable via post (Figure 2b). Patients were instructed during the first study visit to wear the wristband on their nondominant wrist, and because it is not waterproof, they were advised to remove it during heavy exercise, showering, or bathing. Apart from these times, patients were instructed to wear it for 24 hours a day, throughout the 7-day study period.

The wristband is an actigraph designed to record ACC at a sampling rate of 32 Hz (2 G range; 16 bit resolution; Imec Chill band device). ACC signals were used to estimate physical activity during sleep, and an algorithm was developed (incorporating their sleep schedule) to calculate objective sleep variables, which included the following:

1. *SleepEfficiency*_*objective*_: (TST/TIB) × 100 where TST is the total sleep time in minutes according to the ACC signal and TIB (time in bed) is TST plus time in bed not sleeping according to the ACC signal.
2. *SleepOnsetLatency*_*objective*_: time between the patient recording getting into bed and the first registered point of sleep according to the ACC signal.
3. *EarlyMorningAwakening*_*objective*_: time between the last registered point of sleep according to the ACC signal and the patient recording getting out of bed.
4. *WakeAfterSleepOnset*_*objective*_: total duration of awakening during the night, i.e., time between first and last segment of sleep in minutes on ACC.

In clinical practice, a cutoff at 30 minutes is used to indicate prolonged *SleepOnsetLatency* or *WakeAfterSleepOnset* (20), and normal *SleepEfficiency* is considered to be 85%–90% (21).

Periods during which there was no change in arm angle larger than 5° over at least 5 minutes, were classed as bouts of sustained inactivity or potential sleep periods. The sleep period time was calculated using a formula by van Hees et al (22)—only potential sleep periods that fell within the window of sleep period time were considered—see Supplementary Material, http://links.lww.com/AJG/D55 for more details. We also excluded all patients with ≤4 nights of matched ESM and wristband ACC data from the analysis (4).

Patients were also instructed to self-report on their sleep schedule in a daily sleep diary, including 4 timings documented in sequential order: (i) time they got into bed; (ii) time they intended to fall asleep; (iii) time they first awoke; and (iv) time they got out of bed. These timings were used solely to assist with calculation of actigraphy data. Patients also documented their medication, caffeine, and alcohol intake per day–substances known to affect sleep quality (23).

### Descriptive measures

At the end of the 7-day study period, end-of-week questionnaires were automatically emailed to the patient from the electronic system—Castor Electronic Data Capture (24)—designed to assess the following: baseline demographics; IBS severity (IBS Severity Scoring System) (25); GI symptoms (GI Symptom Rating Scale–IBS) (26); depression symptoms (Patient Health Questionnaire) (27); anxiety symptoms (Generalized Anxiety Disorder–7) (28); GI-specific anxiety (Visceral Sensitivity Index) (29); Quality of life (Short Form-36) (30); sleep quality (PSQI) (31); and sleep disorder screen (Sleep Disorder Symptom Checklist-25); (32). All questionnaires ask participants to report symptoms retrospectively, such that they reflect the same period over which the MEASuRE app was used. The PSQI score is a validated retrospective measure of sleep disturbance whereby patients with scores >5 or >8 can be regarded as having poor sleep quality. See Supplementary Methods, http://links.lww.com/AJG/D55 for full details on the aforementioned questionnaires.

### Statistical analysis

For all data except for cross-lagged panel analysis, R Studio, version April 1, 1106 (2009–2021, RStudio, Posit, PBC) was used to perform analysis. Continuous outcomes are presented as mean (SD) where normally distributed and median (interquartile range [IQR]) where not normally distributed.

ESM questionnaire data was summarized at the individual day level (i.e., the mean of repeated measurements within the day) for abdominal pain, anxiety, depression, and all subjective sleep quality variables. Lower-GI (LGI) symptoms were calculated as the sum of 4 items: gas, borborygmi, bloating, and urge and then summarized at the individual day level. Similarly, upper-GI (UGI) symptoms were calculated as the sum of 4 items: nausea, belching, heartburn, and satiety and then summarized at the individual day level. ESM analysis includes all patients who completed at least one-third of the questions over the 1-week period (i.e., at least 23 of 70) as has been common standard in ESM protocols (33,34).

Cross-lagged panel models were built to investigate the following a priori defined associations: (i) *SleptWell* and GI symptoms (abdominal pain, UGI symptoms, and LGI symptoms)—see Figure 4; (ii) *SleepEfficiency*_*objective*_ and GI symptoms (abdominal pain, UGI symptoms, and LGI symptoms); (iii) *SleepOnsetLatency*_objective_ & anxiety; (iv) *EarlyMorningAwakening*_*objective*_ & depression, while controlling for all autoregressive coefficients (35) (i.e., the extent to which scores on variable X at time point N predict scores on variable X at time point N+1)—see Supplementary Figure 1, http://links.lww.com/AJG/D53. In addition, the following associations were investigated exploratively: *SleptWell* & depression, *SleptWell* & anxiety, *SleepEfficiency*_*objective*_ & depression, SleepEfficiency_*objective*_ & anxiety, *SleepOnsetLatency*_*objective*_ & depression, and *EarlyMorningAwakening*_*objective*_ & anxiety. In all cross-lagged analyses, we controlled for the association between sleep and age by including age as a predictor of the sleep measure at each time point (Figure 4). Because *EarlyMorningAwakening*_*subjective*_ *and SleepOnsetLatency*_objective_ were categorical variables and zero inflated, they were not used in the cross-lagged panel analyses. Cross-lagged panel analysis was performed using SAS software, version 9.4 (2013v, SAS Institute, Cary, NC). See Supplementary Methods, http://links.lww.com/AJG/D55 for more details.

Linear mixed-effect models were used to test the relationship between nocturnal abdominal problems and number of awakenings because both variables were measured on the same night, i.e., with no lag between variables, they cannot be compared using cross-lagged panel analysis. Both variables were z scored within subjects. Models included a random intercept and were corrected for repeated measures using an autoregression correlation structure. A *P* value of ≤ 0.05 was considered statistically significant.

## RESULTS

### Baseline demographics

**Figure 3.**
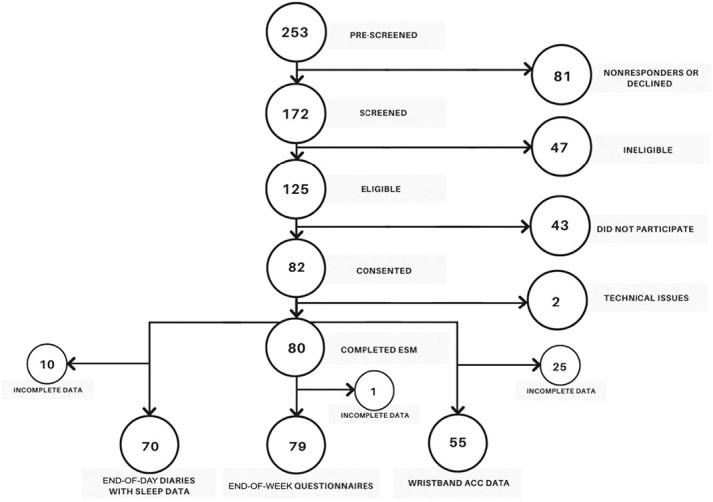
Patient flow chart.

Two hundred fifty-three patients expressed an interest, of whom 80 completed the daily ESM (mean age: 37 [range: 20–68] years, 89% female)—Figure 3. Completion rate of ESM questionnaires was 97.6%, and all patients completed at least one-third of the questions. Approximately 78% (n = 62) of the final cohort were from the community population and 22% (n = 18) from the tertiary clinic. Approximately 22% (n = 17) of patients report taking neuromodulators and 20% (n = 16) were on some form of sedating medication—see Table 1.

**Table 1.**
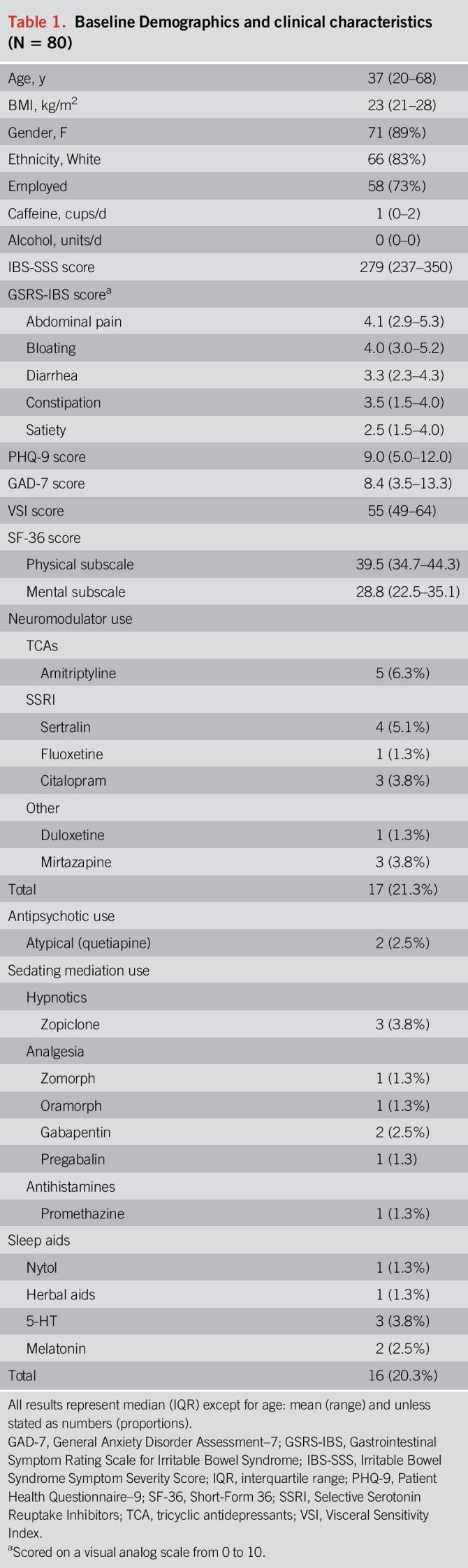
Baseline Demographics and clinical characteristics (N = 80)

### IBS symptoms

The median IBS-Severity Scoring System score indicates moderate severity (279 [IQR: 237–350]). The highest subdomain scores on the GI Symptom Rating Scale–IBS (scored 0–7) were for abdominal pain (median, 4.1 [IQR: 2.8–5.2]) and bloating (median, 4.0 [IQR: 3.0–5.2]). Using ESM (scored 0–10), weekly average group scores tended to be lower than the same symptoms scored on end-of-week questionnaires: abdominal pain (mean: 2.6 [SD: 0.4–4.8]), LGI symptom score (mean, 10.2 [SD: 3.5–16.9]), and UGI symptom score (mean: 7.6 [SD: 2.0–13.2]), which is an observation supported by previous ESM studies.

### Subjective Sleep Quality

Table 2 summarizes the results of subjective sleep quality, recorded each morning by ESM. The median time spent in bed was 9 hours 21 minutes (IQR: 8.7–10.1). Forty-six percent of patients reported a prolonged mean *SleepOnsetLatency*_*su*bjective_ (≥30 minutes) across the week (i.e., they took 30 minutes or longer to fall asleep).

**Table 2.**
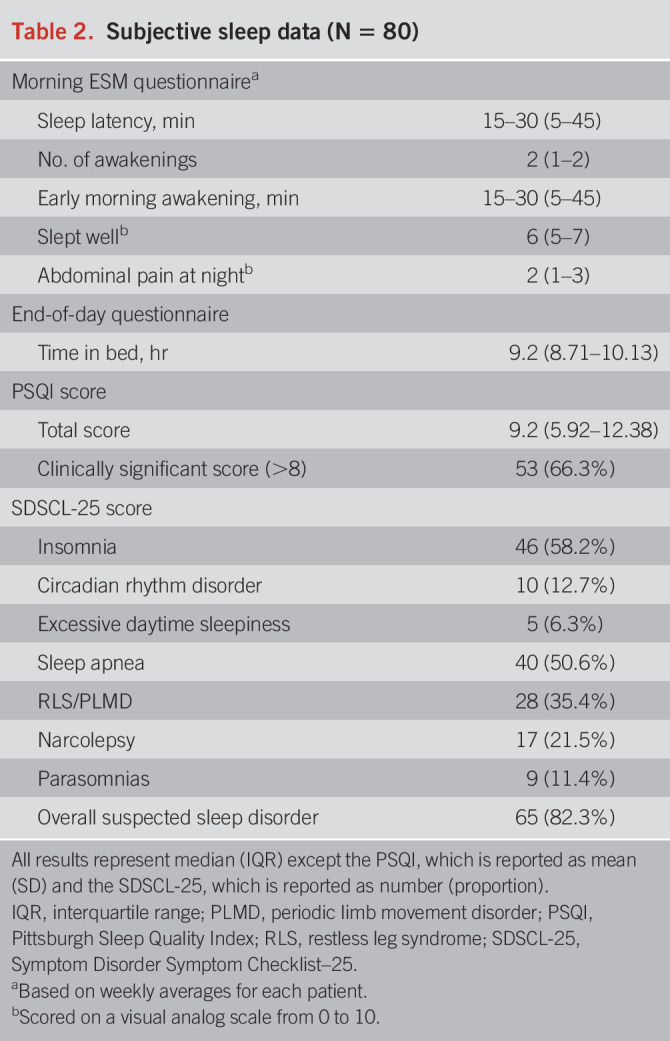
Subjective sleep data (N = 80)

Ninety-five percent had a PSQI ≥ 5 indicating a positive screen for a clinically significant sleep disturbance. Sixty-six percent had a PSQI score ≥ 8, and this may be a more appropriate cutoff for our patient group because it has a greater specificity at this higher cutoff point (36). Twenty-five percent of patients reported reasons for having trouble sleeping during the last month, including both internal reasons (e.g., worry/anxiety/nightmares) and external reasons (e.g., children and pets waking them at night/partners snoring/outside disturbances). Eighty-two percent (n = 65) screened positive for a sleep disorder according to the SDSCL-25 questionnaire, of which the most common disorder was insomnia.

**Figure 4.**
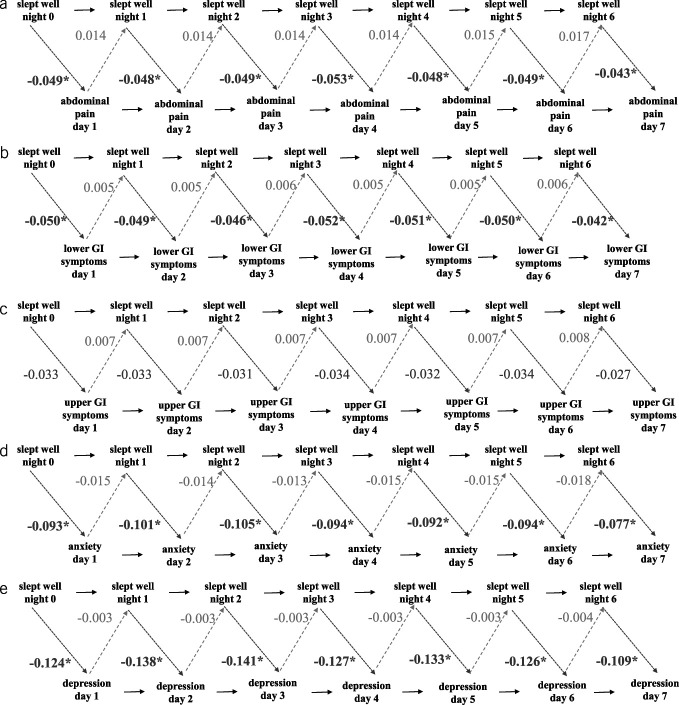
Cross lagged model linking subjective sleep quality with next day GI symptoms. Paths of interest are the cross-lagged paths going from sleep measures at night N to symptoms at day N+1 (dark gray dotted arrow) and from symptoms at day N to sleep measures at night N (light gray dashed arrows). This is investigated while controlling for all auto-regressive coefficients (i.e., stabilities over time, being the extent to which variable X at time T predicts variable X at time T+1, full black arrows). Numbers represent standardized path coefficients. No within-time co-variates since symptoms are day and night. (**a**) Slept well and abdominal pain. (**b**) Slept well and LGI symptoms. (**c**) Slept well and UGI symptoms. (**d**) Slept well and anxiety. (**e**) Slept well and depression. LGI, lower GI; UGI, upper GI. * *P* ≤ 0.05.

In cross-lagged panel analysis, *SleptWell* predicted worse next-day abdominal pain (0.036 < *P* < 0.040), but worse abdominal pain during the day did not predict *SleptWell* during the night (all *P* values = 0.77; Figure 4a). Similarly, *SleptWell* predicted next-day LGI symptoms (0.030 < *P* < 0.032), but worse LGI symptoms during the day did not predict *SleptWell* the following night (all *P* values s = 0.90; Figure 4b). Although the direction of the effect was the same for abdominal pain and LGI symptoms, it was not significant for the UGI symptom model (Figure 4c).

Over the 7-day period, the median number of awakenings was 2 per night (IQR: 1–3) and nocturnal abdominal problems was 2 out of 10 severity (IQR: 0–4). Based on individual *z* scores, an increase of 1 SD in number of awakenings is predicted by an increase of 0.33 SD of nocturnal abdominal problems the same night. Reverse association demonstrated an increase of 1 SD in nocturnal abdominal problems is predicted by an increase of 0.26 SD of number of awakenings the following night. Supplementary Digital Content (see Supplementary Figure 2, http://links.lww.com/AJG/D54) is an effect plot demonstrating the positive association between number of awakenings and nocturnal abdominal problems.

### Objective sleep measures

Fifty-five patients with matched ESM and ACC data were included in the analysis. *SleepEfficiency*_*objective*_ was at the lower end of normal range (median, 86% [CI 95%: 85–87]) and *SleepOnsetLatency*_*objective*_ was at the higher end of normal range (median, 29.9 minutes [IQR: 10.5–60.7]) and 10.8% of patients had a score of zero. *EarlyMorningAwakening*_*objective*_ was minimal (median, 5.5 minutes [IQR: 11.3–40.0]) and 30.7% had a score of zero. *WakeAfterSleepOnset*_*objective*_ was within the normal range (median, 22 minutes [IQR: 11.3–40.0]).

We found no significant cross-lagged paths in the relationship between *SleepEfficiency*_*objective*_ and (i) abdominal pain (ii) LGI symptoms and (iii) UGI symptoms (see Supplementary Figure 1, http://links.lww.com/AJG/D53). We found that prolonged *SleepOnsetLatency*_*objective*_ predicted *less* anxiety the next day (0.008 < *P* < 0.012), while anxiety during the day did not predict *SleepOnsetLatency*_*objective*_ the following night (all *P*'s = 0.76). We found no significant cross-lagged paths between *EarlyMorningAwakening*_*objective*_ and next-day depression (see Supplementary Figure 1, http://links.lww.com/AJG/D53).

### Exploratory cross-lagged associations

Outside of the main research questions, associations between anxiety and depression, on the one hand, and sleep quality and efficiency, on the other hand, were explored using cross-lagged analysis. *SleptWell* negatively predicted next-day depression (*P* < 0.001) and anxiety (*P* < 0.001) but not vice versa (*P* = 0.95 and *P* = 0.74, respectively), indicating that better subjective sleep quality was associated with less next-day depressive and anxiety symptoms—see Figure 4d,e. No significant associations were found between *SleepEfficiency*_*objective*_ and depression or anxiety nor between *SleepOnsetLatency*_*objective*_ and depression or *EarlyMorningAwakening*_*objective*_ and anxiety.

## DISCUSSION

We present the first study to measure the relationship between real-time reported GI symptoms and sleep quality in patients with IBS, using both subjective and objective sleep measures. Our cohort of patients with moderately severe IBS reported high levels of sleep disturbance (PSQI), and most of the patients met criteria for at least 1 sleep disorder (SDSCL-25). Our study demonstrates first, that poor subjective sleep quality predicted next-day abdominal pain, but the converse was not true. Second, poor subjective sleep quality predicted next-day higher scores for abdominal pain, LGI symptoms, depression, and anxiety, but the converse was not true. Last, objectively measured sleep quality did not predict next-day abdominal symptom severity.

Sleep quality over the previous month, based on PSQI scores, seemed to be slightly worse for our cohort compared with other studies reporting sleep disturbance in IBS (3). Having sampled the largest IBS cohort in a sleep study to date, our study may be a more accurate representation. This was the first study to use the SDSCL-25 score in an IBS cohort and found that a high percentage of our group screened positive for a sleep disorder. The identification of individual sleep disorders is useful to target treatment options; however, the scoring system for the SDSCL-25 is highly sensitive, e.g., participants who self-report snoring on 3 or more days per week screen positively for obstructive sleep apnea on the SDSCL-25, which is not the only criterion used clinically to diagnose this sleep disorder. It is recommended that results of the SDSCL-25 score should be interpreted within the clinical context of the individual patient (32).

Poor subjective sleep quality predicts next-day abdominal pain, but the reverse relationship was not significant. This aligns with previous IBS sleep studies (3), although they did not assess for reverse causality in a single model controlling for the other direction. It is well known that chronic pain and sleep are related in a bidirectional manner, whereby comorbidity results in greater symptom severity and more disability (37). There is evidence from longitudinal studies to support the notion that sleep disturbance is a stronger, more reliable predictor of pain than pain is of sleep disturbance (38). For instance, in a study that also used cross-lagged panel models, increases in monthly insomnia severity ratings were associated with next-month increases in average daily pain in temporomandibular disorder, but not vice versa (37). In one study, the absence of nocturnal abdominal symptoms as an additional symptom marker has been found to increase the performance of Rome III diagnostic criteria in making a positive diagnosis of IBS (38). Our finding of low levels of nocturnal abdominal symptoms supports this association and may explain the low level of nocturnal awakening in our patients.

Potential common pathways for the effect of sleep on pain include dysregulation of the hypothalamic pituitary adrenal axis, which is a known pathophysiological mechanism in IBS (39) and is associated with disrupted sleep (40). One study concluded that poor self-reported sleep quality was significantly associated with greater cortisol reactivity (i.e., increases from baseline) after an acute physical stressor, i.e., cold pressor task (41). Autonomic nervous system dysregulation, as measured by heart rate variability, has also been demonstrated in patients with IBS (42) and is linked to poor sleep efficiency (43). Orr et al (44) found that patients with IBS have greater sympathetic activity during waking and greater overall sympathetic dominance during rapid eye movement sleep compared with healthy controls. Last, sensitization of the central nervous system is an established mechanism of chronic pain in IBS, and recent studies suggest that low-grade neuroinflammation, resulting from sleep disturbance, has a role to play in the establishment and/or maintenance of central sensitization (45).

We observed a unidirectional relationship between poor subjective sleep quality and abdominal pain, LGI symptoms, depression, and anxiety the following day, which contrasts slightly with the findings of Buchanan et al (4) who found that self-reported sleep quality significantly predicted next-day abdominal pain and anxiety, but not GI symptoms nor depression. Differences could be accounted for by: lower mean symptom scores, smaller sample size (n = 24), and use of different sleep measurement tools (end-of-day diaries) compared with our real-time measurements. Our finding that objectively measured sleep quality did not predict next-day symptoms highlights the important role of perception in sleep-symptom relationships.

In our study, the relationship between anxiety and *SleepOnsetLatency*_*objective*_ was converse to our expectations, i.e., prolonged *SleepOnsetLatency*_*objective*_ predicted less anxiety the next day. This implies that staying up in bed but not sleeping has the potential to reduce next-day anxiety and could indicate that relaxing activities performed in bed such as reading, journaling, or talking to a partner may help to reduce next-day anxiety, which future qualitative studies should evaluate. People often overestimate their sleep onset latency and underestimate their total sleep time, relative to objective measures, and reasons for this include: inability to distinguish being awake from early sleep stages; worry/anxiety; selective attention toward sleep-related threats, and the presence of brief awakenings (46). Therefore, future studies will need to consider these factors as possible contributors to the finding observed in our study.

We conclude that better subjective sleep quality predicts next-day depression, but not vice versa. Although a significant relationship between early morning awakening and depression is highlighted in the literature, to date, no studies have examined this relationship in the population with IBS. Future research should seek to confirm this finding while examining the potential mechanisms of action for this finding.

Strengths of this study include recruitment of a relatively large number of community patients with IBS diagnosed according to Rome IV criteria (78% of our cohort, n = 62), who are likely to be representative of the UK population with IBS, making our findings highly generalizable. Moreover, there were exceptionally high completion rates of ESM, and we were able to simultaneously assess both directions of the sleep-symptom relationship by virtue of using cross-lagged panel models. Furthermore, sleep efficiency was used as the main objective sleep measure, which is the most widely used measure of sleep quality (47,48) and ought to be replicated as a primary outcome in future studies on sleep and IBS to enable easy comparison between findings.

Limitations of our study include first, subjective sleep variables—*SleepOnsetLatency*_*subjective*_ *and EarlyMorningAwakening*_*subjective*_—were measured as ordinal variables, and response categories crossed the clinically significant threshold of 30 minutes. This was because the ESM questions were designed for measuring IBS symptoms in a multicenter study, not with sleep measurement in mind. Future studies should consider these variables as continuous to avoid issues with interpretation. Second, it must be noted that our algorithm for ACC interpretation is at best an estimation of sleep/wake states. Actigraphy is unable to detect the difference between awake vs sleep as accurately as PSG; hence, our output of *SleepOnsetLatency*_*objective*_ and *EarlyMorningAwakening*_*objective*_ had high percentages of zero values and could not be easily transformed. Ideally, we would like to have access to PSG to validate our findings. Third, IBS subtype analyses were not performed because of the limited sample size, and previous studies examining sleep disorders by IBS subtype found no significant variation between them (49). Moreover, this study was underpowered to control for differences in neuromodulator and sedative use and between the community and the tertiary cohorts. Fourth, the sum of individual symptoms was used to create the UGI and LGI symptom scores; however, this may not be representative of the different contributions of individual symptoms to UGI and LGI symptom burden in daily life. Last, this study was conducted during the COVID-19 pandemic, which has since been demonstrated to have affected both sleep quality (50) and GI symptoms (51) alongside mood symptoms. However, if symptoms were influenced, then these findings simply represent the more severe end of the spectrum and should be interpreted in this context.

The main findings of this study support subjective sleep quality influencing next-day GI symptoms but not vice versa. Objective sleep measures did not predict next-day symptoms, potentially supporting the conclusion that it is the perception of sleep quality that is most influential on next-day abdominal pain. Our findings suggest gastroenterologists should consider sleep as a lifestyle factor influencing GI symptoms, highlighting the role of assessing subjective sleep quality/screening for sleep disorders in the clinical consultation and to guide future research into whether interventions aimed at improving sleep also affect GI symptom severity.

## CONFLICTS OF INTEREST

**Guarantor of the article:** Qasim Aziz, PhD, FRCP.

**Specific author contributions:** R.T., L.V., L.V.O., D.K., and Q.A.: planned the study. R.T. and S.P.: conducted the study and collected the data. R.T., J.C., J.E., H.V., H.F., and M.V.D.H.: interpreted the data. R.T.: drafted the manuscript. All authors edited the draft manuscript to arrive at the final version.

**Financial support:** Funding for the development and validation of the ESM tool was provided by Grunenthal GmbH, Aachen, Germany to Maastricht University.

**Potential competing interests:** D.K. has received research funding from Grunenthal, Allergan, Will Pharma, Rome Foundation, ZonMw, MLDS, UEG, Horizon 2020, Horizon Europe, and speaker's fee from Dr Falk (paid to host institution). R.T., L.V., H.F., S.P., J.C., J.E., L.V.O., M.V.D.H., and Q.A. have no conflicts of interest.

**Study Highlights**

**WHAT IS KNOWN**

✓ Sleep disturbances are more common in patients with irritable bowel syndrome compared with those in healthy subjects.
✓ Sleep quality is an independent factor affecting gastrointestinal (GI) symptoms.

**WHAT IS NEW HERE**

✓ Poor subjective sleep quality predicts next-day abdominal pain and lower-GI symptom scores.
✓ Worse abdominal pain and lower-GI symptom scores do not predict subjective sleep quality the following night.
✓ Objective sleep measures are not associated with next-day GI symptoms.

## Supplementary Material
