# Supplementary material for: Poor Subjective Sleep Quality Predicts Symptoms in Irritable Bowel Syndrome Using the Experience Sampling Method
Source: Am J Gastroenterol. 2023 Sep 22;119(1):155–64. doi: 10.14309/ajg.0000000000002510 (PMC10758350; doi:10.14309/ajg.0000000000002510)
Supplement: Supplementary file 1 [file acg-119-155-s001.pptx]

## Slide 1
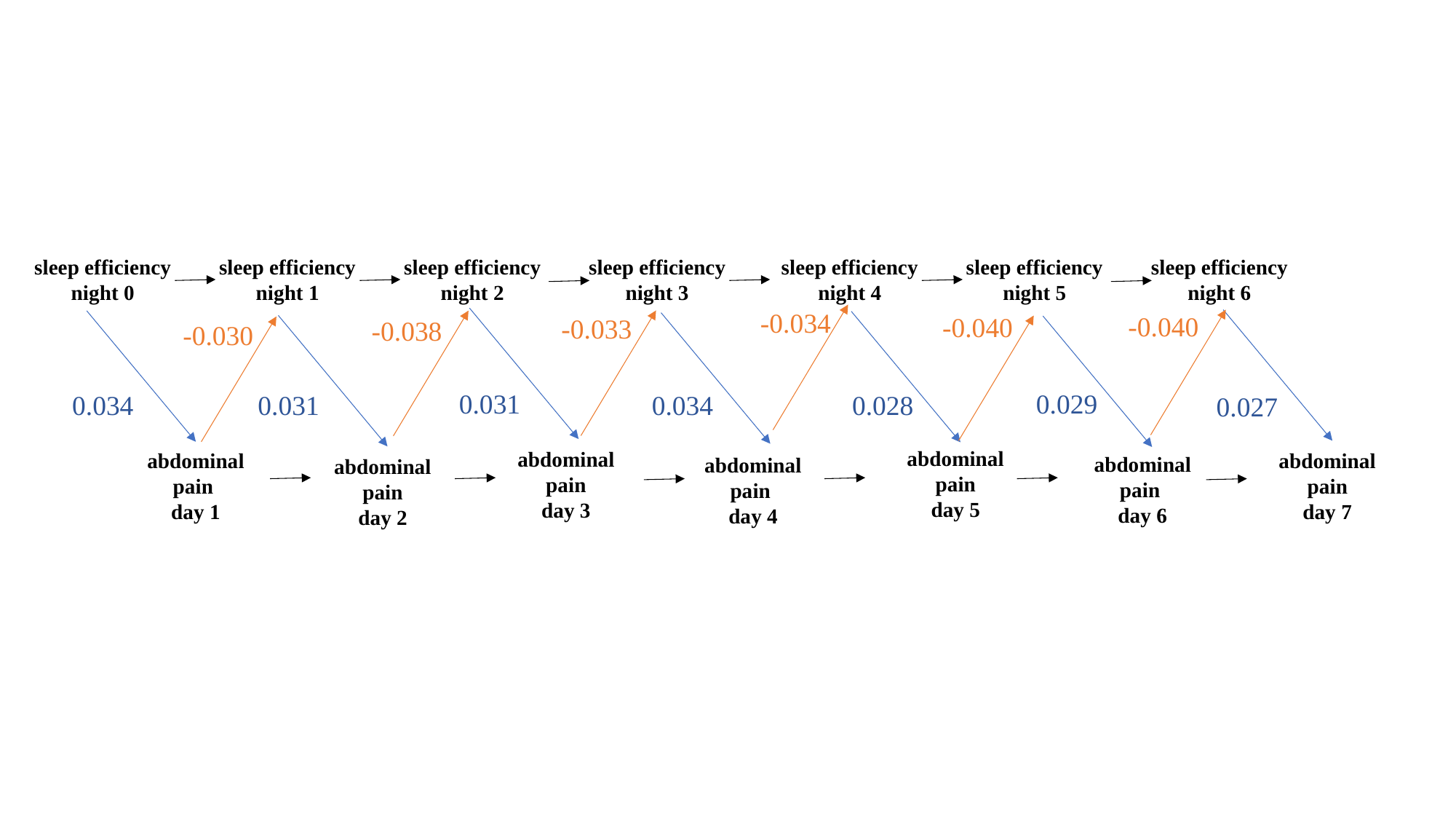

sleep efficiency night 0
sleep efficiency night 1
sleep efficiency night 2
sleep efficiency night 3
sleep efficiency night 4
sleep efficiency night 5
sleep efficiency night 6
-0.034
-0.040
-0.040
-0.033
-0.038
-0.030
0.031
0.029
0.034
0.031
0.034
0.028
0.027
abdominal pain
day 5
abdominal pain
day 3
abdominal pain
day 1
abdominal pain
day 7
abdominal pain
day 6
abdominal pain
day 4
abdominal pain
day 2

## Slide 2
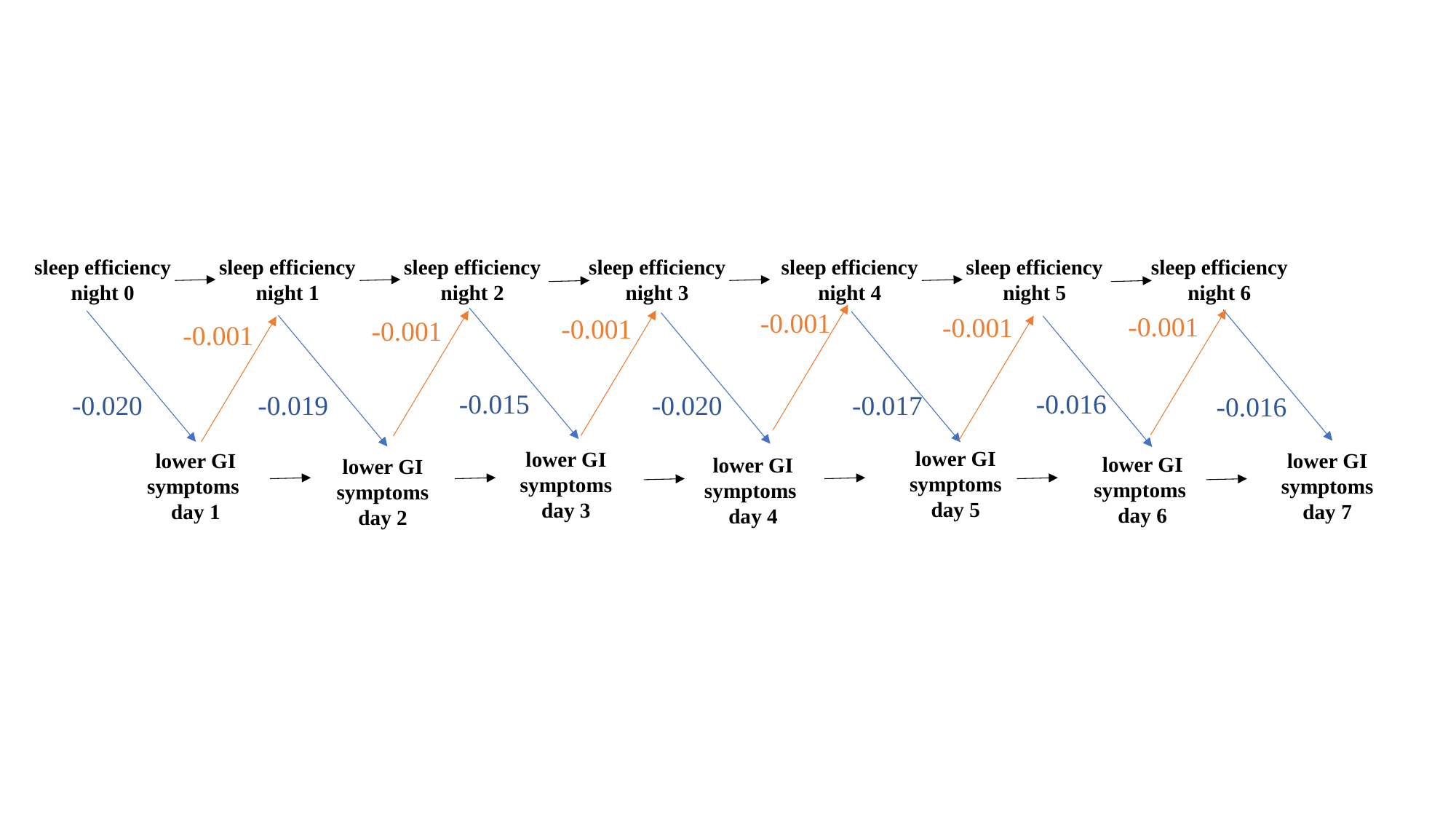

sleep efficiency night 0
sleep efficiency night 1
sleep efficiency night 2
sleep efficiency night 3
sleep efficiency night 4
sleep efficiency night 5
sleep efficiency night 6
-0.001
-0.001
-0.001
-0.001
-0.001
-0.001
-0.015
-0.016
-0.020
-0.019
-0.020
-0.017
-0.016
lower GI symptoms
day 5
lower GI symptoms
day 3
lower GI symptoms
day 1
lower GI symptoms
day 6
lower GI symptoms
day 4
lower GI symptoms
day 2
lower GI symptoms
day 7

## Slide 3
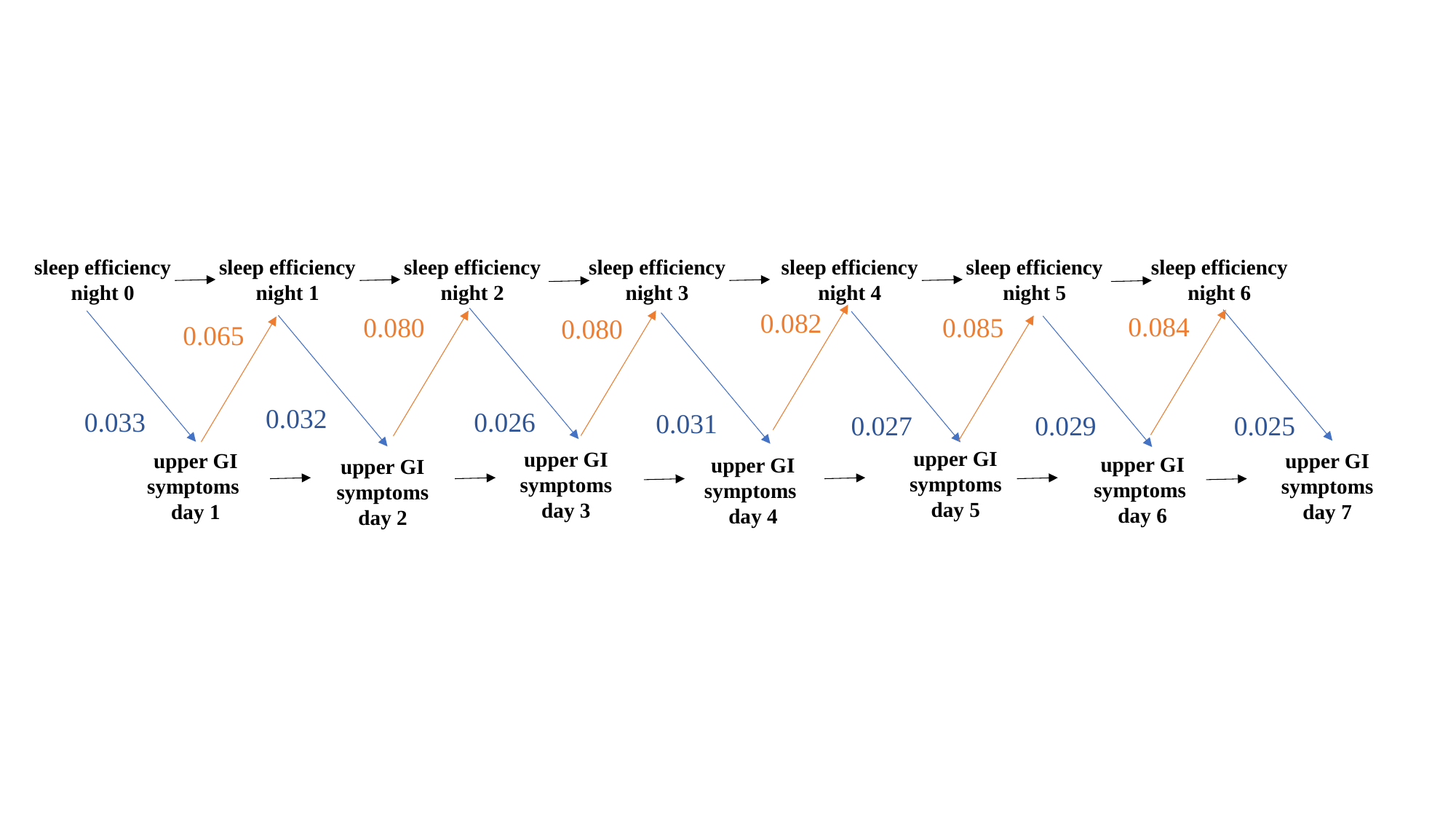

sleep efficiency night 0
sleep efficiency night 1
sleep efficiency night 2
sleep efficiency night 3
sleep efficiency night 4
sleep efficiency night 5
sleep efficiency night 6
0.082
0.084
0.080
0.085
0.080
0.065
0.032
0.033
0.026
0.031
0.029
0.027
0.025
upper GI symptoms
day 5
upper GI symptoms
day 3
upper GI symptoms
day 1
upper GI symptoms
day 6
upper GI symptoms
day 4
upper GI symptoms
day 2
upper GI symptoms
day 7

## Slide 4
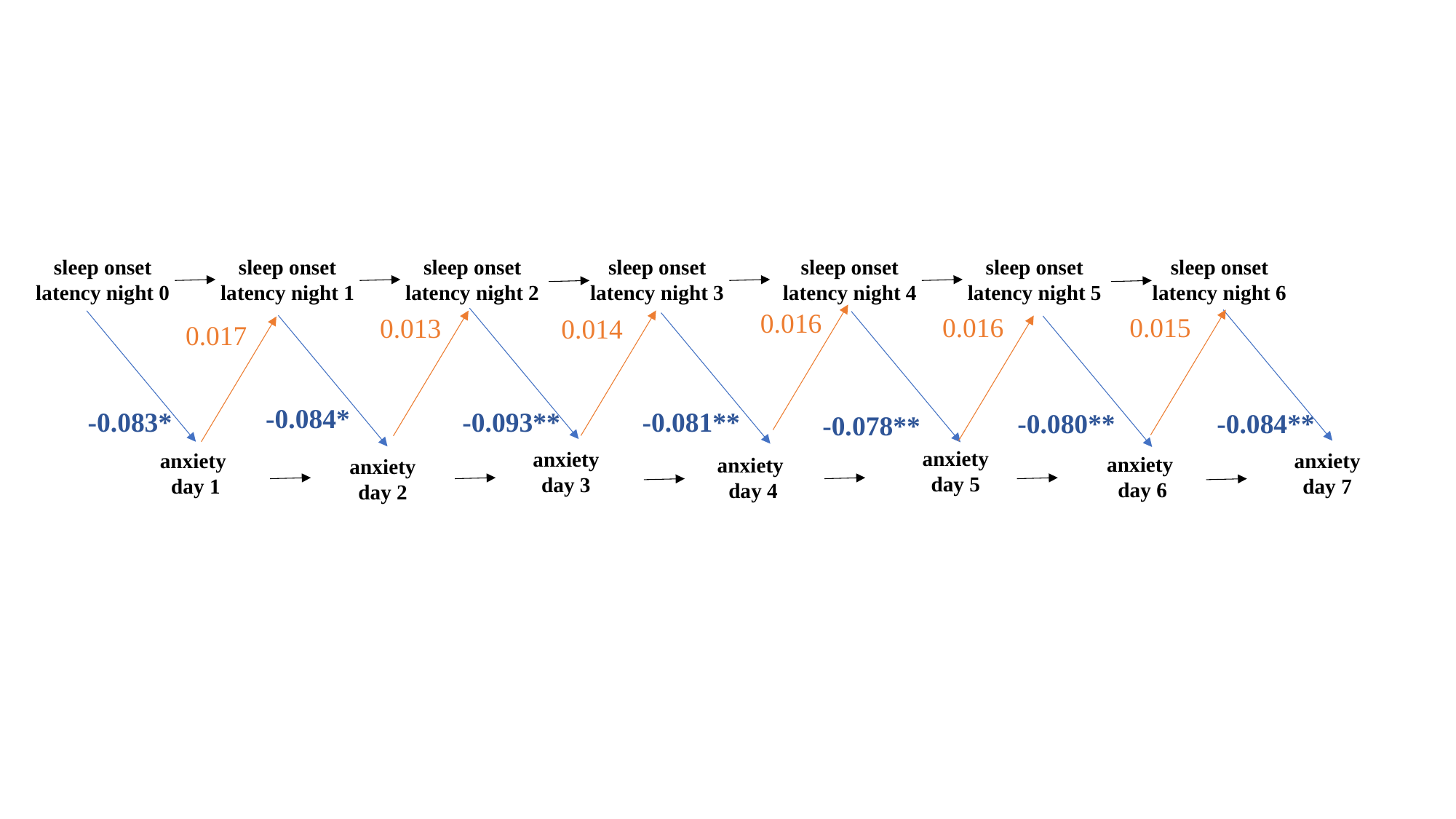

sleep onset latency night 0
sleep onset latency night 1
sleep onset latency night 2
sleep onset latency night 3
sleep onset latency night 4
sleep onset latency night 5
sleep onset latency night 6
0.016
0.015
0.016
0.013
0.014
0.017
-0.084*
-0.081**
-0.083*
-0.093**
-0.080**
-0.084**
-0.078**
anxiety
day 5
anxiety
day 3
anxiety
day 1
anxiety
day 7
anxiety
day 6
anxiety
day 4
anxiety
day 2

## Slide 5
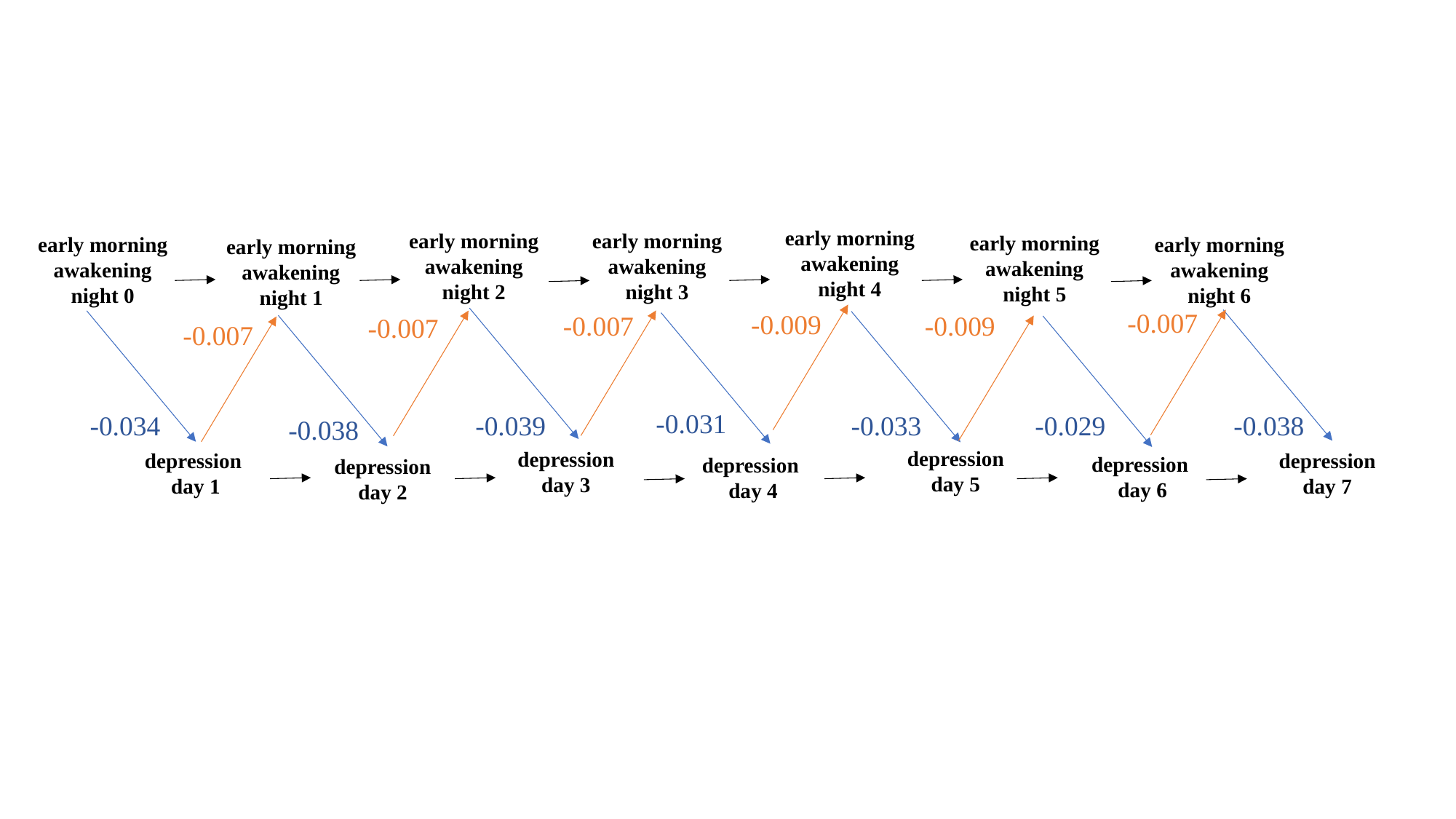

early morning awakening night 4
early morning awakening night 2
early morning awakening night 3
early morning awakening night 5
early morning awakening night 6
early morning awakening night 0
early morning awakening night 1
-0.007
-0.009
-0.009
-0.007
-0.007
-0.007
-0.031
-0.029
-0.033
-0.039
-0.034
-0.038
-0.038
depression
day 5
depression
day 3
depression
day 1
depression
day 6
depression
day 4
depression
day 2
depression
day 7

## Slide 6
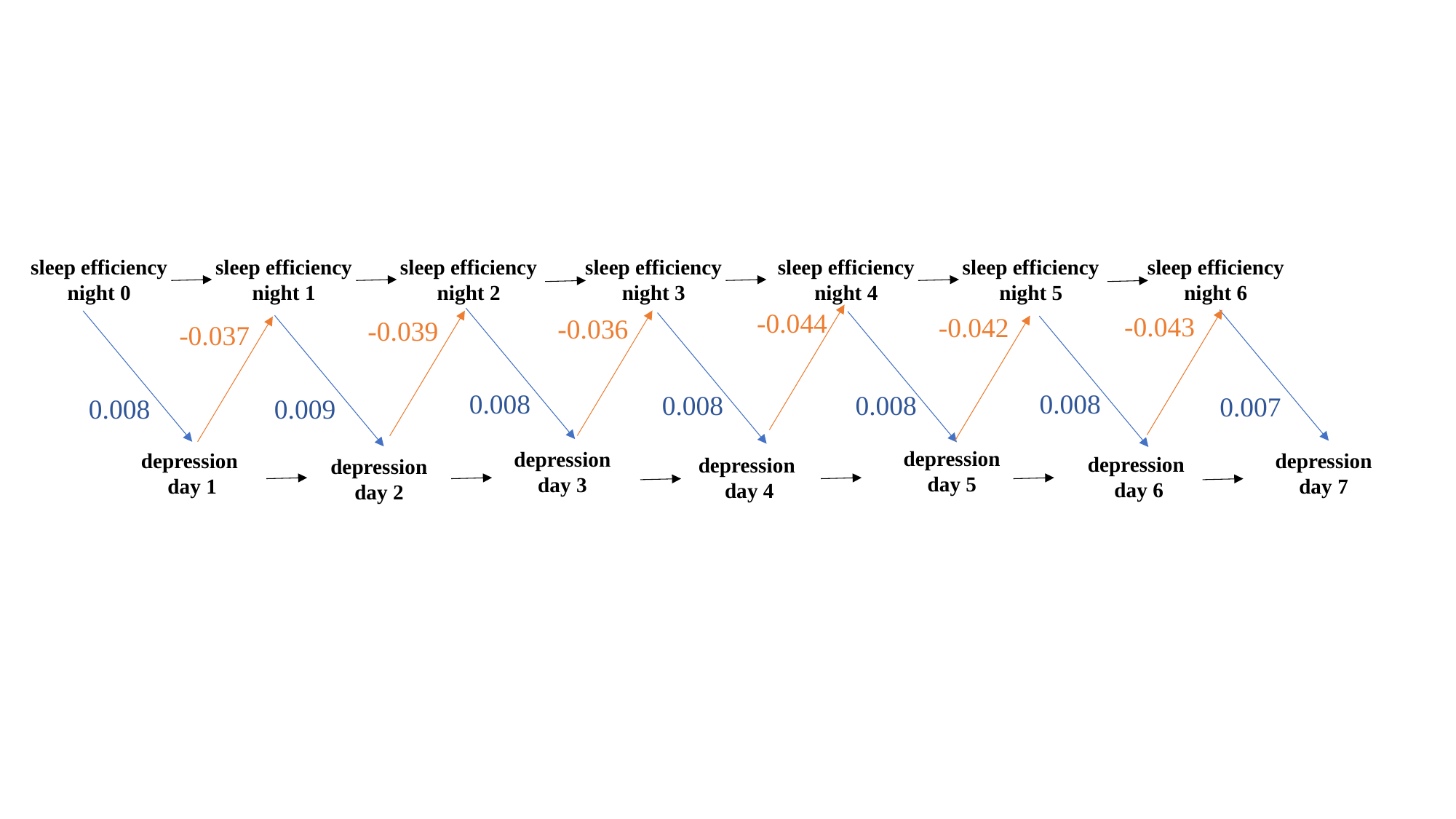

sleep efficiency night 0
sleep efficiency night 1
sleep efficiency night 2
sleep efficiency night 3
sleep efficiency night 4
sleep efficiency night 5
sleep efficiency night 6
-0.044
-0.043
-0.042
-0.036
-0.039
-0.037
 0.008
 0.008
 0.008
 0.008
 0.007
0.008
0.009
depression
day 5
depression
day 3
depression
day 1
depression
day 6
depression
day 4
depression
day 2
depression
day 7

## Slide 7
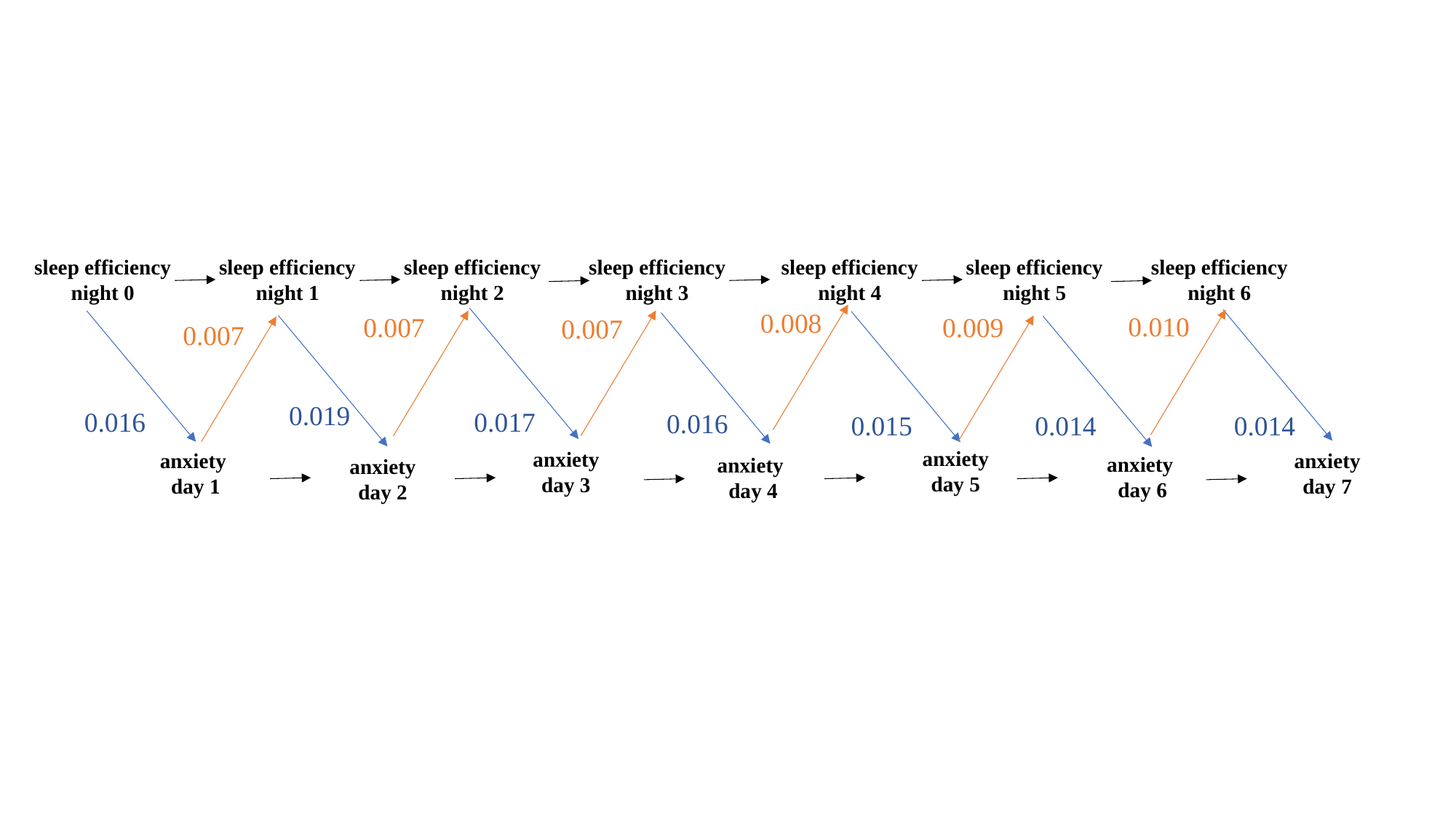

sleep efficiency night 0
sleep efficiency night 1
sleep efficiency night 2
sleep efficiency night 3
sleep efficiency night 4
sleep efficiency night 5
sleep efficiency night 6
0.008
0.010
0.007
0.009
0.007
0.007
0.019
0.016
0.017
0.016
0.014
0.015
0.014
anxiety
day 5
anxiety
day 3
anxiety
day 1
anxiety
day 6
anxiety
day 4
anxiety
day 2
anxiety
day 7

## Slide 8
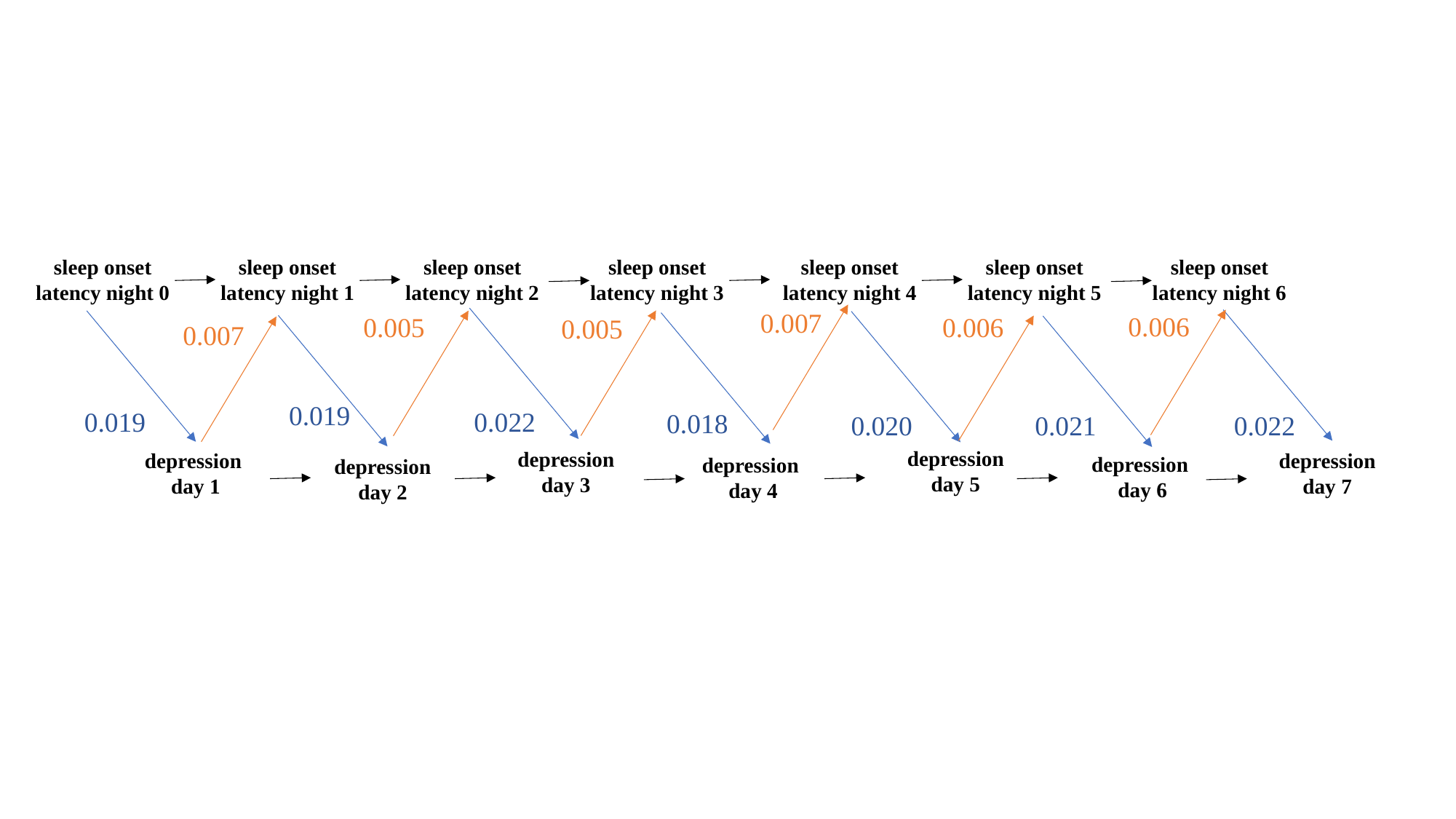

sleep onset latency night 0
sleep onset latency night 1
sleep onset latency night 2
sleep onset latency night 3
sleep onset latency night 4
sleep onset latency night 5
sleep onset latency night 6
0.007
0.006
0.005
0.006
0.005
0.007
0.019
0.019
0.022
0.018
0.021
0.020
0.022
depression
day 5
depression
day 3
depression
day 1
depression
day 6
depression
day 4
depression
day 2
depression
day 7

## Slide 9
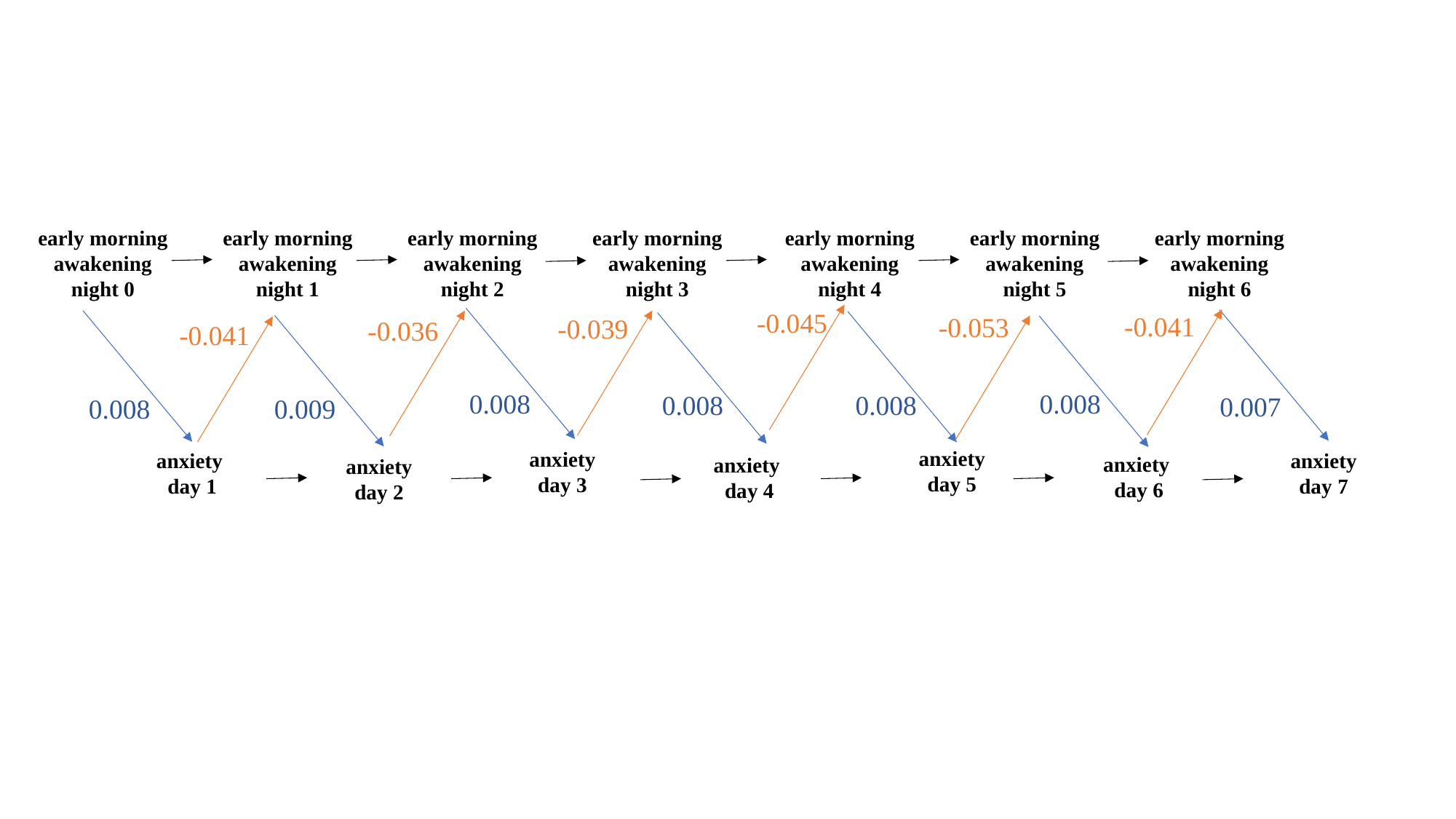

early morning awakening night 0
early morning awakening night 1
early morning awakening night 2
early morning awakening night 3
early morning awakening night 4
early morning awakening night 5
early morning awakening night 6
-0.045
-0.041
-0.053
-0.039
-0.036
-0.041
 0.008
 0.008
 0.008
 0.008
 0.007
0.008
0.009
anxiety
day 5
anxiety
day 3
anxiety
day 1
anxiety
day 6
anxiety
day 4
anxiety
day 2
anxiety
day 7
