# Supplementary figures and images for: Poor Subjective Sleep Quality Predicts Symptoms in Irritable Bowel Syndrome Using the Experience Sampling Method
Source: Am J Gastroenterol. 2023 Sep 22;119(1):155–64. doi: 10.14309/ajg.0000000000002510 (PMC10758350; doi:10.14309/ajg.0000000000002510)

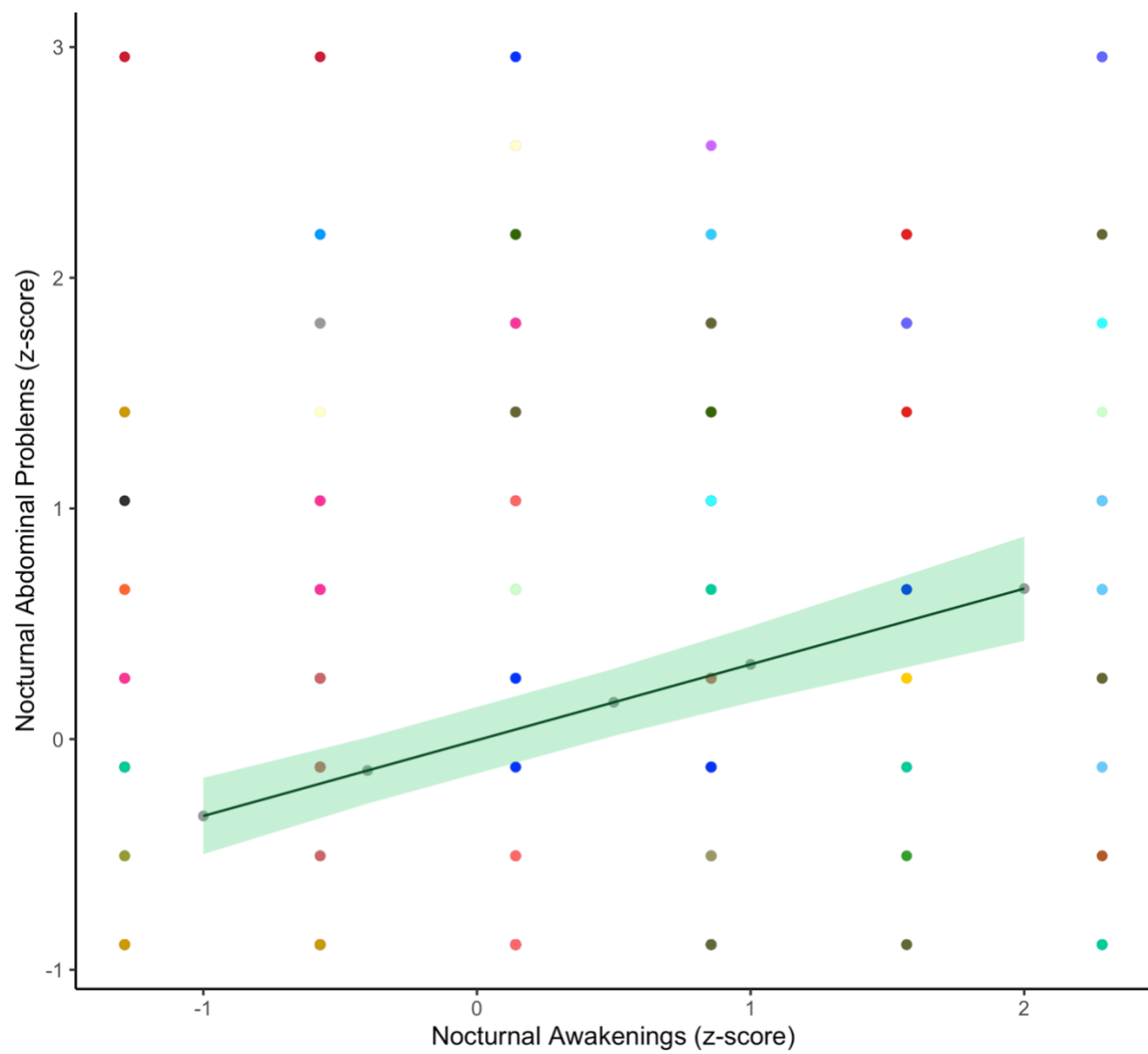

Supplement: Supplementary file 2 [file acg-119-155-s002.pdf]
