# Supplementary material for: Poor Subjective Sleep Quality Predicts Symptoms in Irritable Bowel Syndrome Using the Experience Sampling Method
Source: Am J Gastroenterol. 2023 Sep 22;119(1):155–64. doi: 10.14309/ajg.0000000000002510 (PMC10758350; doi:10.14309/ajg.0000000000002510)
Supplement: Supplementary file 3 [file acg-119-155-s003.docx]

**Figure Legends**

**Figure 1: study design.** V1: visit 1. V2: visit 2. ESM: electronic sampling method. ACC: accelerometer.

**Figure 2: A: screenshot of the ESM question for the assessment of abdominal pain; B: wristband** (3D accelerometer).

**Figure 3: patient flow chart.**

**Figure 4: cross lagged model linking subjective sleep quality with next day GI symptoms.** Paths of interest are the cross-lagged paths going from sleep measures at night N to symptoms at day N+1 (blue arrows) and from symptoms at day N to sleep measures at night N (orange arrows). This is investigated while controlling for all auto-regressive coefficients (i.e. stabilities over time, being the extent to which variable X at time T predicts variable X at time T+1, full black arrows). Numbers represent standardised path coefficients. No within-time co-variates since symptoms are day and night. A: slept well and abdominal pain. B: slept well and lower gastrointestinal (GI) symptoms. C: slept well and upper gastrointestinal (GI) symptoms. D: slept well and anxiety. E: slept well and depression. *p≤ 0.05 (colour)

**Figure 4: cross lagged model linking subjective sleep quality with next day GI symptoms.** Paths of interest are the cross-lagged paths going from sleep measures at night N to symptoms at day N+1 (dark grey dotted arrow) and from symptoms at day N to sleep measures at night N (light grey dashed arrows). This is investigated while controlling for all auto-regressive coefficients (i.e. stabilities over time, being the extent to which variable X at time T predicts variable X at time T+1, full black arrows). Numbers represent standardised path coefficients. No within-time co-variates since symptoms are day and night. A: slept well and abdominal pain. B: slept well and lower gastrointestinal (GI) symptoms. C: slept well and upper gastrointestinal (GI) symptoms. D: slept well and anxiety. E: slept well and depression. *p≤ 0.05 (black and white)

**Supplementary figure 1: Cross lagged model linking objective sleep variables and next day GI symptoms.** Paths of interest are the cross-lagged paths going from sleep measures at night N to symptoms at day N+1 (blue arrows) and from symptoms at day N to sleep measures at night N (orange arrows). This is investigated while controlling for all auto-regressive coefficients (i.e. stabilities over time, being the extent to which variable X at time T predicts variable X at time T+1, full black arrows). Numbers represent standardised path coefficients. No within time co-variates since symptoms are day and night. A: sleep efficiency and abdominal pain. B: sleep efficiency and lower gastrointestinal (GI) symptoms. C: sleep efficiency and upper gastrointestinal (GI) symptoms. D: sleep onset latency (SOL) and anxiety. E: early morning awakening (EMA) and depression. F: sleep efficiency and depression. G: sleep efficiency and anxiety. H: sleep onset latency (SOL) and depression. I: early morning awakening and anxiety. *p≤ 0.05, **p≤ 0.005 (colour)

**Supplementary figure 1: Cross lagged model linking objective sleep variables and next day GI symptoms.** Paths of interest are the cross-lagged paths going from sleep measures at night N to symptoms at day N+1 (dark grey dotted arrow) and from symptoms at day N to sleep measures at night N (light grey dashed arrows). This is investigated while controlling for all auto-regressive coefficients (i.e. stabilities over time, being the extent to which variable X at time T predicts variable X at time T+1, full black arrows). Numbers represent standardised path coefficients. No within time co-variates since symptoms are day and night. A: sleep efficiency and abdominal pain. B: sleep efficiency and lower gastrointestinal (GI) symptoms. C: sleep efficiency and upper gastrointestinal (GI) symptoms. D: sleep onset latency (SOL) and anxiety. E: early morning awakening (EMA) and depression. F: sleep efficiency and depression. G: sleep efficiency and anxiety. H: sleep onset latency (SOL) and depression. I: early morning awakening and anxiety. *p≤ 0.05, **p≤ 0.005 (black and white)

**Supplementary figure 2: An effect plot demonstrating the positive association between nocturnal awakenings and nocturnal abdominal problems scored by ESM on the same night.** Each colour represents a different subject. Nocturnal abdominal problems variable transformed using boxcox transformation and both variables were z-scored. (colour)

**Supplementary figure 2: An effect plot demonstrating the positive association between nocturnal awakenings and nocturnal abdominal problems scored by ESM on the same night.** Nocturnal abdominal problems variable transformed using boxcox transformation and both variables were z-scored. (black and white)

# Supplementary material

Supplementary methods

The wristband ACC data allows estimation of sleep by the position of the patient’s arm, relative to the horizontal plane. We resampled the ACC data from 32Hz to a single point every 5 seconds (0.2Hz). Each data point has the median of the Z angle perpendicular to the skin surface using the formula below (52).


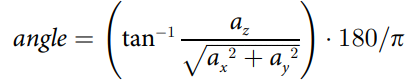


The algorithm by Cole et al (53) was applied on the sleep estimation and Webster’s rules (54) were applied to the outcome. A quality indicator was used to assess the non-wear time within each SPT window (i.e. poor quality indicated by the sum of the mean difference G values of each axis within a five minute window less than 0.003G). If less than 70% of the SPT window was poor quality, it was discarded from the analysis.

IBS-SSS is a composite score to reflect real-time GI symptoms, each measure is rated 0 to 100 with total scores ranging from 0 to 500 (mild; 75-174, moderate; 175-299, severe 300-500). GSRS-IBS comprises five GI symptom domains (abdominal pain, bloating, diarrhoea, constipation, and satiety) each rated on a 1-7 scale, with a recall period of one week. PSQI is a composite score of sleep quality on a 1-4 scale with a recall period of one month, where a threshold of ≥ 5 is taken to indicate a clinically significant sleep disturbance. SDS-CL-25 is a screen for sleep disorders on a 0-4 scale, where a symptom score of 4 (>3 x week) is taken to be consistent with a positive screen for a sleep disorder, with a recall period of 3 months - results are presented as percentages at the group level.

No within-time correlations were controlled for since symptoms reflect symptoms during the day and sleep variables reflect sleep at night. All variables were boxcox transformed before performing the analysis. Auto-regressive coefficients were fixed over time (i.e. the strength of the relationship between variable X at day n and day n+1 equals the strength of the relationship between variable X at day n+1 and day n+2). Three variations of the cross-lagged structural equation model were tested: 1) cross-lagged path coefficients fixed over time; 2) cross-lagged path coefficients unfixed over time, 3) cross-lagged path coefficients going from day 2 to night 2 and from night 1 to day 2 fixed over time, but allowed to differ from cross-lagged paths going from day 1 to night 1 and from night 0 to day 1. Modification indices to improve model fit were requested from the output for all 3 models and auto-regressive paths that were sensical (i.e. going forward in time) were added to the models. Model fit (Chi-square, RMSEA, Bentler’s CFI, AGFI) between the 3 adjusted models was compared; the best fitting model was further adjusted based on modification indices for auto-regressive paths until adding auto-regressive paths would no longer lead to improvements in model fit.
